# Self-Monitoring of Balance Performance can Reduce the Rate of Falls Among Older Adults

**DOI:** 10.3389/fspor.2021.680269

**Published:** 2021-09-24

**Authors:** Katharine E. Forth, Charles S. Layne, Stefan I. Madansingh

**Affiliations:** ^1^Zibrio Inc., Houston, TX, United States; ^2^Department of Health and Human Performance, University of Houston, Houston, TX, United States; ^3^Center for Neuromotor and Biomechanics Research, University of Houston, Houston, TX, United States; ^4^Center for Neuro-Engineering and Cognitive Science, University of Houston, Houston, TX, United States

**Keywords:** balance, fall risk analysis in older people, postural stability (postural control), older adult, balance health

## Abstract

**Background:** 29% of older adults fall annually, resulting in the leading cause of accidental death. Fall prevention programs typically include exercise training and self-monitoring of physical activity has a positive effect on the self-efficacy and self-regulation of exercise behaviors. We assessed if self-monitoring of fall risk, without an intervention, impacts fall rates.

**Methods:** Fifty-three older adults had open access to a balance measuring platform which allowed them to self-monitor their postural stability and fall risk using a simple 1-min standing balance test. 12-month retrospective fall history was collected and a monthly/bimonthly fall log captured prospective falls. Participants had access to self-monitoring for up to 2.2 years. Fall history and fall incidence rate ratios and their confidence intervals were compared between the periods of time with and without access to self-monitoring.

**Results:** A 54% reduction in the number of people who fell and a 74% reduction in the number of falls was observed when participants were able to self-monitor their postural stability and fall risk, after normalizing for participation length. Further, 42.9% of individuals identified as having high fall risk at baseline shifted to a lower risk category at a median 34 days and voluntarily measured themselves for a longer period of time.

**Discussion:** We attribute this reduction in falls to changes in health behaviors achieved through empowerment from improved self-efficacy and self-regulation. Providing older adults with the ability to self-monitor their postural stability and intuit their risk of falling appears to have modified their health behaviors to successfully reduce fall rates.

## Introduction

Each year, 28.7% of older adults fall in the US resulting in 300,000 hip fractures, over $50 billion dollars in medical costs (Florence et al., 2018), and is the leading cause of accidental death in this population (Gillespie et al., 2012). Many risk factors, including both extrinsic and intrinsic factors, have been identified as related to falling in older adults, including: cognitive impairment, use of sedatives, balance and gait abnormalities, disabilities of the lower limbs, foot problems (Tinetti et al., 1988), vison impairment (Lord and Dayhew, 2001), fall history (Ganz et al., 2007), and fear of falling (Young and Mark Williams, 2015). Environmental hazards and the accidents they cause represent the greatest contributors to falls and are cited as the cause of 30–50% of all falls (Rubenstein, 2006). Rubenstein (2006) explains, however, that intrinsic weaknesses stemming from age and disease or loss of function provide an increased susceptibility to hazards, making older adults more vulnerable to environmental challenges to their postural stability (Rubenstein, 2006).

While some risk factors are permanent, many risk factors can be modulated and these are targeted in fall prevention programs. In fact, a number of lifestyle health behaviors, particularly exercise, are known to influence and reduce the risk of falling (Shumway-Cook et al., 1997; Taggart, 2002; Grabiner et al., 2012; Halvarsson et al., 2015; Shier et al., 2016; Sherrington et al., 2017). Yet, less than half of the older adult population exercise sufficiently for general health benefits, and that number is further reduced for those aged 75 and older (Schoenborn et al., 2013). This deficiency of physical activity in older adults presents a clear opportunity for reducing the likelihood of falling. In fact, many fall prevention interventions include a form of exercise as a major component of their program. As a result, interventions focused on changing exercise health behaviors may also provide an opportunity for changing health behaviors beneficial to fall preventing a fall, such as increasing one's awareness of balance health and fall risk.

Current literature identifies self-efficacy and self-regulation (Bandura, 1991; Lee et al., 2007, 2008) as key components for creating positive health behavior changes in exercise intervention programs. Lee et al. (2008) identify knowledge of “performance accomplishments” and one's “physiological state” as two of the four information sources required for older adults to achieve self-efficacy and overcome psychological barriers to exercise. Self-monitoring that provides feedback and information relating to a particular behavior, has been found to empower individuals in exercise initiation and adherence for a positive effect on exercise health behaviors (Michie et al., 2009). Currently, wearable and connected technologies have made self-monitoring possible for a variety of lifestyle and health metrics. The self-monitoring offered by pedometers, for example, have created positive change in health behaviors and have increased physical activity (Wang et al., 2016; Maher et al., 2017; Sullivan and Lachman, 2017).

Given the overlap between fall prevention and exercise interventions, we hypothesize that providing knowledge of balance performance through unconstrained and longitudinal self-monitoring of postural stability as an indicator of fall risk will serve as a behavioral intervention to reduce the observed rate of falls in older adults.

Presently, there is no at-home method for self-monitoring fall risk and there is limited capacity for fall risk testing within the clinical setting. In this study, we provided residents at a senior living facility and older adults who regularly visit a community center for seniors access to easy-to-use technology which measures postural stability as an indicator of fall risk and tracked them longitudinally to determine if self-monitoring influenced their rate of falls.

## Methods

Fifty-three older adults (age 80.4 ± 10.4 years) (mean ± SD) were recruited from two locations: an independent Senior Living Facility (SLF), *n* = 30 (25 female), and a Senior Community Center (SCC), *n* = 23 (18 female). Individuals were unable to participate if they could not stand for 60 s unassisted or if they self-reported a history of neurodegenerative, vestibular or balance related disease, or had experienced a significant musculoskeletal injury within the last 6 months which limited their ability to stand unassisted. The experimental protocol was approved by the University of Houston Institutional Review Board (IRB), and informed consent was obtained prior to participation.

**Figure 1:**
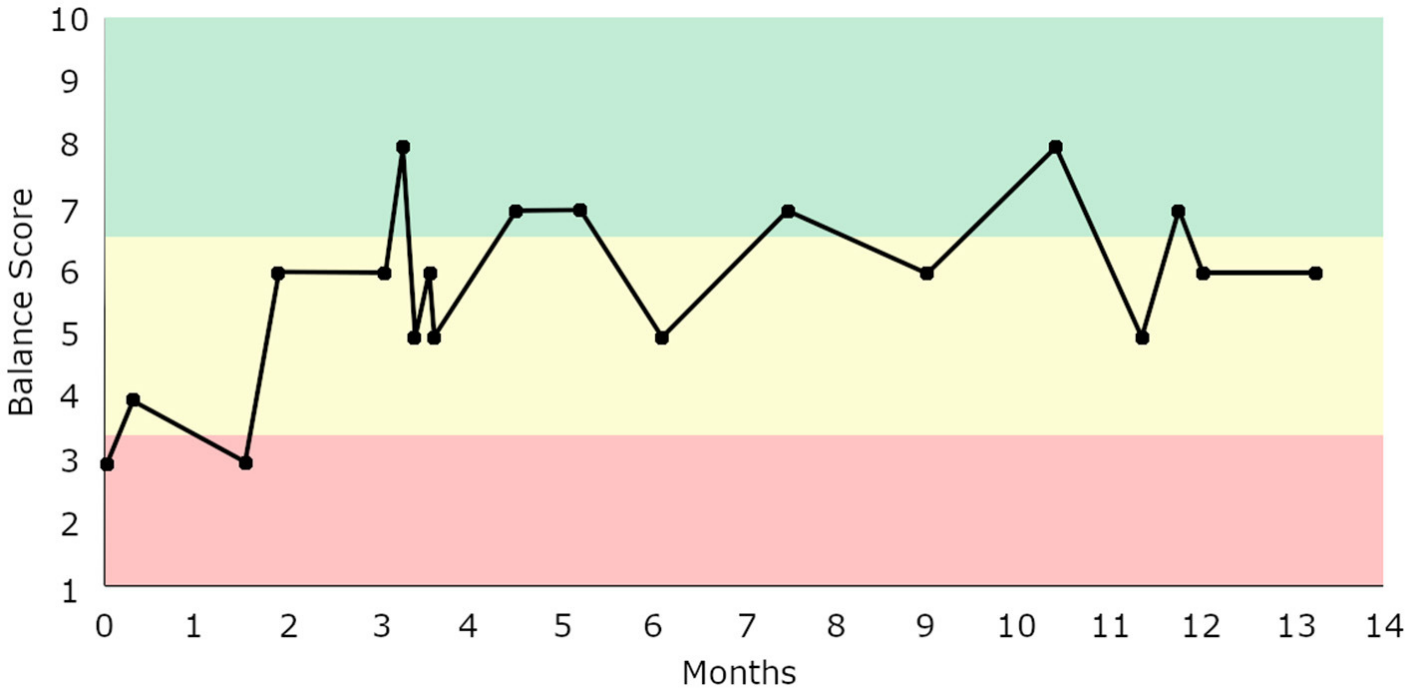
Balance score tracking for a representative older adult who self-selected to assess their postural stability 18 times over the span of 14 months. This individual transitioned from initially scoring in the “high risk” zone (1–3) to comfortably staying within the “moderate risk” zone (4–6), occasionally entering the “low risk” zone.

Each participant performed an eyes-open test of quiet stance on a laboratory-grade force plate (AMTI, Watertown, MA, USA) for 60 s. Participants were instructed to stand comfortably, with their arms to their sides and their eyes forward. Participants were also instructed to stand as still as they possibly could, without moving or talking. Center of pressure (COP) was captured at 100 Hz on the force-plate. Custom software (Labview 2011, National Instruments, TX, USA) was used to calculate a score (from 1 to 10) from the COP data, reflecting an estimate of the participant's postural stability and was referred to as a “balance score.” Briefly, this custom software collected the COP data then performed linear quantifications of postural sway, including: path length, velocity, acceleration and jerk, in both anterior-posterior and medial-lateral directions, as well as non-linear measures of postural stability characterized using a Hidden Markov Model (Rasku et al., 2008; Joutsijoki et al., 2019) were utilized as factors (Forth and Lieberman Aiden, 2019) to calculate the balance score. A score of 1 suggested the lowest stability and 10 suggested the highest stability. The numeric score was displayed on a nearby computer display after the test with an associated color denoting fall risk category. Scores ranging 1–3 indicated high fall risk coinciding with a “red” risk group; scores ranging 4–6 indicated moderate fall risk, coinciding with a “yellow” risk group and scores ranging 7–10 indicated low fall risk coinciding with a “green” risk group (Forth et al., 2020). This “balance score” was immediately made available to the participant upon completion of the test and placed within the context of visual imagery depicting less independence and increased frailty for scores in the “red” risk group and increased independence and low frailty for scores in the “green” risk group. Participants were assured that no categorization was permanent and encouraged to test themselves regularly to track their progress, see Figure 1 for a representative sample.

The testing equipment that provided a postural stability (“balance”) score was available to the SLF for 2.2 years and could be operated at their discretion during this time. The number of tests performed by each participant was recorded. The SCC participants were given three opportunities to test themselves across 6 months, resulting in an approximately bimonthly testing schedule. The duration of prospective monitoring was calculated as the difference between the first test date and the last test date for each individual, representing their participation in person-years (Young, 2005).

Six and 12 month fall history was recorded at the first test and a fall log was obtained via monthly (SLF) or bimonthly (SCC) follow-up phone interview for the duration of study participation (Hannan et al., 2010). A fall event was counted if the participant confirmed that they had “unintentionally reached the ground or a lower level” (Tinetti et al., 1988) during follow-up interviews. Falls resulting from episodes of syncope caused by orthostatic intolerance, or other factors, were not considered balance related falls and were not counted in this study.

To quantify the impact of self-monitoring and knowledge of balance performance upon rates of falls, incidence rate ratios (IRR) and their 95% confidence intervals were calculated to compare: the rate of past falls [*IRR(falls)*] and the observed rate of prospective (future) falls, as well as the number of past fallers with the number of participants who experienced a fall during the prospective portion of the study [*IRR(fallers)*]. A percentage reduction was also calculated comparing the number of past fall events with the number of observed fall events occurring after access to self-monitoring of postural stability (“balance score”) was made available. Incidence and percentage reductions were additionally calculated for all individuals who completed at least 6 and 12 months of self-monitoring. These estimates provided the ability to monitor trends in the calculated IRR for those participants who had monitored time equaling the retrospective period.

The equations for calculating the total person-years for the retrospective and prospective fall data are below:


$$\begin{array}{l} \sum person|years_{retro}=n\;\\ \;\sum person|years_{pros}=\overset{n}{\underset{i=1}{\sum }}duration\;of\;prospective\;monitoring\\ \;=\overset{n}{\underset{i=1}{\sum }}k_{\frac{daysEnrolled}{365}} \end{array}$$

The calculations for the fall rate incidence for retrospective (IR_retro_) and fall rate incidence for the prospective (IR_pros_) are listed before for both fall incidence and number of fallers.


$$\begin{array}{l} \;\#falls_{retro}=\;\text{number of retrospective fall events}\\ \;\#falls_{pros}=\text{number of prospective fall events}\\ IR_{retro}\left(\#falls\right)=\frac{\#falls_{retro}}{\sum person|years_{retro}}\\ \;IR_{pros}\left(\#falls\right)=\frac{\#falls_{pros}}{\sum person|years_{pros}} \end{array}$$

The calculation for the incidence rate ratio (IRR) for fall incidence is below.


$$\begin{array}{l} IRR\left(falls\right)=\frac{IR_{pros}\left(\#falls\right)}{IR_{retro}\left(\#falls\right)} \end{array}$$

The IRR confidence intervals were calculated as a two-tailed 95% limit, using the normalized number of prospective falls (*#falls*_*prosNorm*_) relative to retrospective falls.


$$\begin{array}{l} \;\#falls_{prosNorm}=\#falls_{pros}\;\cdot \;\frac{\sum person|years_{retro}}{\sum person|years_{pros}}\\ 95\%\;CI\;limit\;for\;IRR\left(falls\right)=e^{\left(\ln\left(IRR\right)\;\pm 1.96\sqrt{\frac{1}{\#falls_{retro}}\;+\;\frac{1}{\;\#falls_{prosNorm}}}\;\right)} \end{array}$$

The same calculations were repeated for the number of individuals who fell.


$$\begin{array}{l} \;\#fallers_{retro}=\text{number of participants with retrospective fall events}\\ \;\#fallers_{pros}=\text{number of participants with prospective fall events}\\ IR_{retro}\left(\#fallers\right)=\frac{\#fallers_{retro}}{\sum person|years_{retro}}\\ \;IR_{pros}\left(\#fallers\right)=\frac{\#fallers_{pros}}{\sum person|years_{pros}} \end{array}$$

The calculation for the incidence rate ratio (IRR) for incidence of fallers is below.


$$\begin{array}{l} IRR\left(\#fallers\right)=\frac{IR_{pros}\left(\#fallers\right)}{IR_{retro}\left(\#fallers\right)} \end{array}$$

The IRR confidence interval was calculated for a two-tailed 95% limit, using a normalized number of prospective falls (*#fallers*_*prosNorm*_) relative to retrospective falls.


$$\begin{array}{l} \;\#fallers_{prosNorm}=\#fallers_{pros}\;\cdot \;\frac{\sum person|years_{retro}}{\sum person|years_{pros}}\\ 95\%\;CI\;limit\;for\;IRR=e^{\left(\ln\left(IRR\right)\;\pm 1.96\sqrt{\frac{1}{\#fallers_{retro}}\;+\;\frac{1}{\;\#fallers_{prosNorm}}}\;\right)} \end{array}$$

To estimate the reduction of fall events and reduction in the number of fallers observed in this population after awareness of balance performance made available, retrospective fall history was compared with the normalized prospective fall results to produce a percent reduction:


$$\begin{array}{l} \;Percent\;reduction_{falls}=100\;\cdot \;\frac{\left(\#falls_{retro}\;-\#falls_{prosNorm}\;\right)}{\#falls_{retro}}\\ Percent\;reduction_{fallers}=100\;\cdot \;\frac{\left(\#fallers_{retro}\;-\#fallers_{prosNorm}\;\right)}{\#fallers_{retro}} \end{array}$$

For assessing changes to risk category, the baseline score was defined as the balance score obtained during the initial assessment. A fall risk shift was defined as a change in risk categorization from baseline risk to the risk category associated with the median score of all subsequent scores.

## Results

The total, 53 person-years for the retrospective fall history was collected in this study. The total number of person-years for the prospective fall data was 38.61, as not all participants were tracked for a full year. The SCC participants had shorter tracked periods as they only had three opportunities for testing. The SLF participants, on the other hand, tested themselves 22.3 times on average (ranging from 3 to 107 self-initiated measurements). 96.6% of all SLF participants tested themselves monthly, of which 75.9% tested weekly, biweekly or triweekly. The remaining SLF participants tested at a frequency of 2–3 months. During the prospective observation, no participant fell more than once, therefore #falls_pros_ and #fallers_pros_ are numerically equivalent. Table 1 provides demographics and fall events for all included participants stratified by location.

**Table 1:** Demographics (mean ± SD) and fall events of the participants tracked, stratified by location.

	**All sites**			**Senior living facility (SLF)**			**Senior community center (SCC)**		
	**Female**	**Male**	**Total**	**Female**	**Male**	**Total**	**Female**	**Male**	**Total**
*N*	43	10	53	25	5	30	18	5	23
Age, yrs.	80.1 (±9.4)	81.5 (±7.3)	80.4 (±10.2)	87.5 (±5.0)	82.8 (±8.3)	86.7 (±6.0)	69.8 (±5.9)	80.1 (±7.4)	72.1 (±4.8)
#falls_retro_ (6 m)	16	2	18	11	2	13	5	0	5
#falls_retro_ (12 m)	30	7	37	24	7	31	6	0	6
#fallers_retro_ (6 m)	13	1	14	9	1	10	4	0	4
#fallers_retro_ (12 m)	18	3	21	13	3	16	5	0	5
^†^#falls/ #fallers_pros_	7	0	7	4	1	5	2	0	2
Length tracked, yrs.	0.73 (±0.7)	0.71 (±0.6)	0.73 (±0.6)	1.00 (±0.7)	0.88 (±0.7)	0.98 (±0.8)	0.36 (±0.3)	0.55 (±0.5)	0.4 (±0.3)

† *Denotes the number of falls observed and the number of fallers observed prospectively was equal as no participant fell more than once during the period of enrollment*.

The IR and IRR for the number of falls events and the number of people who fell are listed in Table 2. The true rate ratio lies between 0.13 and 0.53 (all fall events), and 0.21 and 0.98 (number of people who fell), with 95% confidence. These results indicate a reduction in both the total number of fall events and the number of people who fell while self-monitoring their postural stability. The reduction corresponded to a 74.0% reduction in falls and a 54.3% reduction in the number of people who fell after normalizing the rate of prospective falls to the total number of person-years tracked. The trend of reduced falls was further demonstrated with participants who had actual monitored time equaling the retrospective fall history period, Table 3. The reductions in these subsets of the sample ranged from 16.7 to 44.4%.

**Table 2:** Incidence rate (IR) and incidence rate ratio (IRR) results for number of falls and number of people who fell during retrospective analyses and prospective observation.

**Fall events (falls)**	
IR_retro_ (falls)	0.698
IR_pros_(falls)	0.181
IRR [95%CI]	0.260 [0.128–0.528]^*^
**Number of fallers (fallers)**	
IR_retro_(fallers)	0.396
IR_pros_(fallers)	0.181
IRR [95% CI]	0.438 [0.213–0.982]^*^

* *Indicates statistical significance at p < 0.05 (95% confidence interval of IRR excludes null value)*.

**Table 3:** Comparison of number of falls and fallers data for participants with 6 and 12 months of actual fall tracking data with their equivalent fall history.

**Time**	***N***	**Age,yrs**.	**#falls_**retro**_**	**#falls_**pros**_**	**% reduction**	**#fallers_**retro**_**	**#fallers_**pros**_**	**% reduction**
6 months	21	85.5(±6.9)	7	5	28.6%	6	5	16.7%
12 months	13	83.8(±5.8)	9	6	44.4%	6	5	16.7%

42.9% of participants who were initially categorized as “high risk” (scoring 1–3 during their first postural stability test) “shifted” to a lower risk category (entered “moderate” or “low” risk categories), with a median time of 34 days, while participating in this study. Those who shifted participated twice as long as those who remained high risk (participation of those who remained high risk: 209 ± 49.7 days vs. individuals who “shifted”: 441.6 ± 99 days). 23.5% of those who remained high risk experienced a fall event and accounted for 57.1% of all prospective falls observed. In contrast, only 8.3% of those who shifted from a higher to lower risk category experienced a fall. The same fall rate (8.3%) was observed among those who identified as moderate risk at baseline and remained moderate risk throughout the study.

## Discussion

This study is the first to provide aging adults in both independent living facilities and senior community centers access to a self-initiated and self-monitored postural stability measurement, empowering them to take an active role in the monitoring of their balance health. During this study, it was observed that both the number of people who fell and the total number of falls reported were significantly reduced when participants used the self-monitoring scale and received a “balance score” and an estimate of fall risk.

Typically, multifactorial intervention programs are recommended for fall prevention due to the complex nature of falls (Dionyssiotis, 2012). Yet, in the present study no traditional interventions were provided. We attribute the reducing effect on falls to a possible change in health behaviors. If self-monitoring provides an empowering effect through self-efficacy and self-regulation (Bandura, 1991; Lee et al., 2007, 2008; Michie et al., 2009), an objective, quantified measure of fall risk and “balance” could reasonably influence the health behaviors of people aiming to reduce their fall risk – individuals with a history of falling and concern for future fall related injuries.

The greatest reductions in falls were seen in the participants who shifted from high risk at baseline to moderate risk. This “shifting” group indirectly demonstrated increased self-efficacy as they voluntarily measured themselves 232 days longer, on average, than those who remained scoring in the high risk category. It is possible that those self-monitoring for longer were increasing self-efficacy in exercise behaviors, also. By shifting risk category, this group also received positive reinforcement from an improving score, which in and of itself could have been the motivator for continued self-monitoring and behavioral change. Regardless of the cause, the participant's awareness of their fall risk and postural stability state, through knowledge of their “balance score,” may have elicited a change in their health behaviors, which in turn reduced their fall rates, however future studies will be required to determine the nature of this relationship.

Increased exercise, safety measures and “carefulness” are the most likely behavioral changes that would have occurred as they are the easiest for the individual to modulate without clinical assistance. Indeed, exercise alone has demonstrated a protective effect, reducing incidences of falls (Shumway-Cook et al., 1997; Taggart, 2002; Grabiner et al., 2012; Halvarsson et al., 2015; Shier et al., 2016; Sherrington et al., 2017). Changing exercise health behaviors in older adults could be effective as the majority of older adults do insufficient exercise for health benefits (Ashe et al., 2009; Schoenborn et al., 2013). The barriers to initiating and adhering to exercise for many older adults are psychological and attitudinal (Lee et al., 2008). Fortunately, self-monitoring and self-efficacy approaches in exercise interventions have enabled people to overcome these psychological barriers (Lee et al., 2008; Michie et al., 2009) and change health behaviors (Lee et al., 2007, 2008; Michie et al., 2009; Wang et al., 2016; Maher et al., 2017; Sullivan and Lachman, 2017).

In the current study, the effect of self-monitoring may have been further strengthened as the “balance score” provided physiological feedback about the individual's postural stability and balance control—factors which an individual may intuit are related to their risk of falling. This physiological information is not normally accessible with consumer technology thereby providing novel insights into health status in contrast to behavioral measures which are more typically available, such as the number of steps taken in a day. Evidence from a recent fMRI study demonstrated a greater level of brain activation with physiological self-monitoring via blood glucose testing over behavioral self-monitoring through step count estimations, which corresponded with improvements in physical activity health behavior (Whelan et al., 2017a,b). Taken together, it is possible that providing physiological feedback that is less intuitive or less readily observable results in greater health behavior modification. However, any possible impact on an individual's fear of falling may vary based on their fall risk status. Further investigation is necessary to understand the relationship between fear of falling and self-monitoring balance.

Self-monitoring with this physiological feedback may also impact an additional psychological component that is unique to fall prevention: fear of falling. Fear of falling is present in ~21–85% of older adults, fallers and non-fallers alike, and is associated with decreased physical activity, depression, less social contact, avoidance of activities, and increased fall risk (Scheffer et al., 2008). While fear is linked with potential future fall events, the anxiety created by the uncertainty of the individual's risk and likelihood of a fall event is highly noxious (Grupe and Nitschke, 2013). Self-monitoring may also reduce this anxiety by replacing the uncertainty with information about the person's physiological state of stability and fall risk.

We propose that the empowering effect of self-monitoring fall risk and instability could have been especially potent because older adults may have been educated on the benefits of exercise and fall prevention, but they often have limited knowledge and understanding about their own fall risk/balance state. Consequently, the sudden awareness in this domain may have the potential for greater potency than for exercise measures alone, where general knowledge of one's state is greater.

The limitations of this study were related to sources of potential bias in the sample. Serial fallers, individuals who experience recurring falls with minimal injury, have the potential to skew fall rates in aging populations. By comparing the number of fallers in addition to the total number of falls observed, the inflating bias of serial fallers upon the total number of falls is limited and helps to provide confidence in a generalizable interpretation of the results. The sample may also have bias due to the smaller sample and strong gender bias. The gender bias is difficult to avoid due to the gender ratio of females to males in this age group. Females are generally more receptive and proactive about preventive health (Center for Disease Control Prevention, 2001), so may have been more likely to voluntarily participate and seek to change their health behaviors. However, despite the small numbers, a reduction in the number of falls within the male population was observed.

To address potential bias of the small sample, we recruited from two different types of locations. The independent living facility included on-site residential living, while the senior community center was only accessible during daytime hours. Consequently, the length of participant follow-up for the purposes of fall tracking was variable, further compounded by participant availability and dropout due to loss of life. Furthermore, the increased follow-up with individuals in the independent living facilities (monthly) may have encouraged greater participation than those in the community (follow-up every 3 months). To explore the influence of variable follow-up lengths, an assessment of a smaller subset of participants who had actual tracked months equal to the fall history durations was performed at 6 and 12 months of tracking. Again, the reduced trend in the actual number of falls and the number of fallers was observed at both the 6 and 12 months periods. Across both locations there was a variety of participant lifestyles ranging from single to cohabitation, limited to extensive activity, and international travel to a local daily existence. There was also a significant imbalance between male and female participants at each of the sites, however this is consistent with expectations of heterogenous populations among older adults (Lowsky et al., 2014). Finally, self-reporting of falls may have introduced measurement error as fall events are well-known to be under reported with increasing recall time (Mackenzie et al., 2006). In this study, the longest recall period for remembering fall events was 2 months prospectively and 12 months retrospectively. As a result, it is expected that retrospective fall rates are under-reported in this sample, which would have the consequence of limiting the effect of self-monitoring of balance upon the number of reported falls and fallers.

A final limitation of this study is the fact that participants self-selected the cadence and duration of their participation, as well as self-selected their responses to the balance scores they received. Although this study lacked controlled exploration of factors which may influence balance health, postural stability, and fall risk, we propose that is provides a real-world preliminary evaluation of the impact of objective balance health assessments upon fall risk in a predictably heterogeneous population of older adults (Lowsky et al., 2014).

## Conclusions

This study suggests that the awareness gained from self-monitoring of balance and fall risk has a positive effect on fall rates. Empowering self-assessment for older adults was also observed to reduce the incidence of falls. Although the determination of causal factors associated with this reduction in falls is not addressed in the current study, we expect successful future interventions will leverage self-monitoring of balance as a primary component of care alongside traditional fall-reduction interventions. Future work is needed to further understand the role of gender, and determine if fear of falling, individual self-efficacy, physical activity levels and other health behaviors are altered as a result of self-monitored physiological feedback related to postural instability and fall risk.

## Data Availability Statement

The raw data supporting the conclusions of this article will be made available by the authors, without undue reservation.

## Ethics Statement

The studies involving human participants were reviewed and approved by University of Houston Institutional Review Board (IRB). The patients/participants provided their written informed consent to participate in this study.

## Author Contributions

KF and SM developed the theoretical framework for the study and CL provided guidance to limit conflict of interest. SM developed the experimental framework. KF performed data collection and synthesized results. KF, CL, and SM participated in data interpretation and preparation of the manuscript. All authors contributed to the article and approved the submitted version.

## Funding

Zibrio Inc., a privately held company, provided financial support for this study.

## Conflict of Interest

Zibrio Inc. employs the KF and SM representing a potential for conflict of interest. CL from the University of Houston received no funding support for this study and provided guidance throughout to minimize these conflicts.

## Publisher's Note

All claims expressed in this article are solely those of the authors and do not necessarily represent those of their affiliated organizations, or those of the publisher, the editors and the reviewers. Any product that may be evaluated in this article, or claim that may be made by its manufacturer, is not guaranteed or endorsed by the publisher.
